# Trends in ELISA-Based Flavivirus IgG Serosurveys: A Systematic Review

**DOI:** 10.3390/tropicalmed8040224

**Published:** 2023-04-13

**Authors:** Fatima Ericka S. Vista, Ourlad Alzeus G. Tantengco, Micah D. Dispo, Danna Mae S. Opiso, Christian Luke D. C. Badua, John Patrick Z. Gerardo, Juan Raphael M. Perez, Karol Ann T. Baldo, Day-Yu Chao, Leslie Michelle M. Dalmacio

**Affiliations:** 1 Department of Biochemistry and Molecular Biology, College of Medicine, University of the Philippines Manila, Manila 1000, Philippineslmdalmacio@up.edu.ph (L.M.M.D.); 2 Department of Physiology, College of Medicine, University of the Philippines Manila, Manila 1000, Philippines; 3 Department of Biology, College of Science, De La Salle University, Manila 0922, Philippines; 4 Department of Epidemiology and Biostatistics, College of Public Health, University of the Philippines Manila, Manila 1000, Philippines; 5 College of Medicine, University of the Philippines Manila, Manila 1000, Philippines; 6 Graduate Institute of Microbiology and Public Health, National Chung Hsing University, Taichung 40227, Taiwan; dychao@dragon.nchu.edu.tw

**Keywords:** flavivirus, dengue virus, Zika virus, Japanese encephalitis virus, west Nile virus, IgG ELISA, seroprevalence

## Abstract

Flaviviruses include virus species that are major public health threats worldwide. To determine the immunity landscape of these viruses, seroprevalence studies are often performed using IgG ELISA, which is a simple and rapid alternative to the virus neutralization test. In this review, we aim to describe the trends in flavivirus IgG ELISA-based serosurveys. A systematic literature review using six databases was performed to collate cohort and cross-sectional studies performed on the general population. A total of 204 studies were included in this review. The results show that most studies were performed on dengue virus (DENV), whereas Japanese Encephalitis Virus (JEV) was the least studied. For geographic distribution, serosurveys followed known disease prevalence. Temporally, the number of serosurveys increased after outbreaks and epidemics except for JEV, for which studies were performed to demonstrate the effectiveness of vaccination campaigns. Commercial kits were more commonly used than in-house assays for DENV, West Nile Virus (WNV), and Zika virus (ZIKV). Overall, most studies employed an indirect ELISA format, and the choice of antigens varied per virus. This review shows that flavivirus epidemiology is related to the regional and temporal distribution of serosurveys. It also highlights that endemicity, cross-reactivities, and kit availabilities affect assay choice in serosurveys.

## 1. Introduction

Flaviviruses are a group of virus species that belong to the genus Flavivirus and the family Flaviviridae [1]. Through their mosquito vectors, flaviviruses such as dengue virus (DENV), Zika virus (ZIKV), Japanese encephalitis virus (JEV), and West Nile Virus (WNV) are transmitted to humans and cause disease. They are also among the most important flaviviruses based on occurrence and disease impact [2]. Though there are many medically important flaviviruses that affect humans, DENV, JEV, and WNV are known to be established threats, since they cause the most number of infectious and are widespread globally [3]. Meanwhile, ZIKV is a flavivirus of interest, since its recent re-emergence brought about millions of infections that increased the global distribution of this virus [3,4]. In tropical and subtropical countries, these flaviviruses are major public health threats [5]. However, in recent years, these viral infections have also become a medical concern for temperate regions [6,7,8]. Arboviruses such as Yellow fever virus (YFV), Powassan virus, Usutu virus, tick-borne encephalitis virus, and Ilheus virus, among others, have a more limited geographic distribution and disease burden and are not included in this review.

DENV is a flavivirus spread by the *Aedes* mosquito that causes dengue fever (DF), dengue hemorrhagic fever (DHF), and dengue shock syndrome (DSS) [9]. DENV is endemic in at least 100 countries in Asia, the Pacific, the Americas, Africa, and the Caribbean [10] and causes around 390 million infections yearly [11]. ZIKV shares the same mosquito vector as dengue [12] and similarly presents with non-specific flu-like symptoms [13]. ZIKV infection may lead to chronic disability due to neurologic sequelae [14] and may also cause congenital brain anomalies in infants who contract intrauterine infection [15]. ZIKV is present globally, with the World Health Organization (WHO) reporting that 89 countries have documented mosquito-borne virus transmission [16]. Its disease impact ranges from thousands to millions of infections annually depending on the year [3]. JEV is a mosquito-borne flavivirus transmitted by *Culex* spp. mosquitoes [1]. Among those hospitalized with JE infection, 30% die, and half of survivors end up with severe neurologic sequelae [17]. Global cases of JEV are estimated to be nearly 68,000 each year, with approximately 13,600 to 20,400 deaths [18]. Lastly, WNV belongs to the JEV serocomplex and is also transmitted by *Culex* mosquitoes [19]. Like JEV, most infected people are asymptomatic, with a minority developing a systemic viral illness and less than 1% developing symptoms of meningitis and encephalitis [20]. It is mainly a disease found in North America, the Middle East, Africa, Europe, and Australia that causes less than 10,000 cases reported yearly [3].

Due to the endemicity of these viruses, surveillance systems are recommended for guiding public health policies such as vaccination programs, which are based on disease burden and population immunity. However, this is made difficult by several factors such as the non-specific symptoms and the short viremic period of flavivirus infections [21], as well as the serological cross-reactivity between flaviviruses [22]. As of writing, only DENV and JEV have vaccines that are currently on the market. For DENV, it is the Dengvaxia vaccine, for which pre-vaccination screening is recommended prior to vaccine administration to ensure seronegative individuals are not vaccinated [23]. For JEV, surveillance plays a role in guiding governments in determining the effectiveness of vaccination programs. Further, seroprevalence studies are needed for flavivirus infections. Though a majority of those infected are asymptomatic, disease surveillance continues to depend on reports from clinics, hospitals, and laboratories that deal with symptomatic cases [24,25].

A wide array of methods are used for seroprevalence determination, such as hemagglutination inhibition assays [26], immunofluorescence assays [27,28], microsphere immunoassays [29], ELISA [30], lateral flow immunoassays [31], and virus neutralization tests [32]. Among these, ELISA is most frequently used, since it provides a rapid, simple, safe, high-throughput and relatively inexpensive alternative to the gold standard virus neutralization test [33,34,35]. The principle of ELISA is based on the formation of specific antigen–antibody complexes and their subsequent quantification through an enzyme-induced colorimetric change [36]. ELISA formats can be classified as either direct, indirect, sandwich, or competitive, depending on the order in which antibodies and antigens are added into the assay [36]. Direct ELISAs are known to have the lowest sensitivity, indirect and competitive ELISAs have high sensitivities, and sandwich ELISAs are the most sensitive [36]. However, the choice of format to be used does not depend on sensitivity alone but also on the purpose of detection. For instance, it may depend on whether antigens or antibodies are to be detected and on the availability of capture and detector antibodies for the protein of interest. In virus serology, ELISA can be used to detect virtually any antibody isotype. For flaviviruses, IgG and IgM are usually studied, with IgM as a marker of acute infection and IgG as a marker of past infection. This is why in seroprevalence studies, which usually involve healthy populations, the IgG ELISA is the most commonly performed method [24]. Despite this, the IgG ELISAs used in these studies vary significantly regarding test kits, assay format, and antigens. To our knowledge, a comparison of the IgG ELISA characteristics in different countries and over time has not yet been described.

This review aims to determine the trends in ELISA-based flavivirus IgG serosurveys across different populations and throughout the years. This can help to identify appropriate strategies for population-specific flavivirus serosurveillance to help ensure that assays are used to provide accurate results, minimizing the effect of potential cross-reactivities.

## 2. Materials and Methods

### 2.1. Protocol and Registration

This protocol follows the guidelines set by the Preferred Reporting Items for Systematic Reviews and Meta-Analyses (PRISMA) statement [37]. It is also registered in the International Prospective Register of Systematic Reviews (PROSPERO) database (CRD42022362104).

### 2.2. Eligibility Criteria

Cross-sectional and cohort seroprevalence studies employing IgG ELISA as an immunoassay to analyze serum samples from any healthy human population were included. Studies published in languages other than English were excluded, as well as studies that only involve non-healthy populations (i.e., febrile patients, hospitalized patients, suspected or confirmed cases, or cases of other infections such as but not limited to HIV, malaria, or chikungunya), because they are more likely to be seropositive, and thus their antibody response will not reflect that of the general population. Further, studies were excluded if they were review articles, letters to the editor, or comments; if they were below the average quality score; or if the full text was not available.

### 2.3. Information Sources and Search Strategy

Comprehensive searches of six electronic databases, including EBSCO CINAHL, OVID MEDLINE, the World Health Organization Institutional Repository for Information Sharing (WHO IRIS), the Latin American and Caribbean Health Sciences Database (Lilacs), Scopus, and the Scientific Electronic Library Online (SciELO), were performed including studies published from inception to 2022. The following search strategy was used: ELISA OR “enzyme linked immunosorbent assay” OR “enzyme-linked immunosorbent assay” AND “breakbone fever” OR “break-bone fever” OR dengue OR JEV OR “Japanese Encephalitis*” OR “West Nile Virus” OR “zika*” AND prevalence OR Seroprevalence OR seroepidemio* OR accuracy OR “diagnostic performance” OR sensitivity OR specificity OR AUROC OR “ROC curve” OR “receiver operating characteristic curve”.

### 2.4. Study Selection

The results of the initial search strategy were screened by title and abstract. This was performed independently by eight reviewers. Full-text articles were retrieved for all the eligible studies. Eight reviewers evaluated full-text articles independently to determine whether the articles fulfilled the inclusion and exclusion criteria. All irrelevant articles were excluded, with recordkeeping of the reasons for exclusion.

### 2.5. Data Collection

The following data were collected from each study: study title, first author, year of publication, country of origin, study population, source of ELISA assay (commercial vs. in-house), and the specific brand, if commercially available, the virus of interest, ELISA format, and antigen used. Only data from IgG ELISA assays using serum specimens were included. If other immunoassays, antibody isotypes, or serum specimens were also used in the study, data for these were not collected. Moreover, only data from healthy populations were extracted if studies included both healthy and non-healthy populations.

### 2.6. Quality Assessment

For quality assessment and to evaluate the risk of bias in each study, the Joanna Briggs Institute (JBI) Critical Appraisal Checklist for Prevalence Studies was used [38]. Two authors did this independently, with discrepancies resolved by a third study team member. Each question in the nine-item checklist was answered with either “yes”, “no”, “unclear”, or “not applicable”. For each study, a score was calculated based on the number of questions answered with a “yes”. A study was deemed representative of the target population if it belonged to the general population of the geographic area being studied. Participant recruitment was considered appropriate if random sampling was performed. The sample size was considered adequate if it fulfilled the minimum target sample size computed by the authors. If no calculation was performed, but population characteristics were given, sample size adequacy was assessed based on the expected sample size. The validity of methods, standardization of measurements, and statistical analyses were evaluated based on the described methodology of each study. The adequacy of sample coverage and the response rate were evaluated based on the number of samples tested for IgG ELISA. Articles were given an overall appraisal of “include”, “exclude”, or “seek further info” based on the reviewers’ judgment. Reasons for exclusion were noted. The quality assessment of screened articles is shown in Table S1.

### 2.7. Statistical Analysis

Data gathering was focused on the methodology portion of each paper. Based on this, we compared the immunoassays based on the virus of interest, the type of ELISA assay (whether commercial or in-house), ELISA format (indirect, direct, dot blot ELISA, etc.), and antigens used. Trends over the years and across countries for these variables were noted. All analyses were performed in R v.4.2.2 [39].

## 3. Results

### 3.1. Study Selection

**Figure 1:**
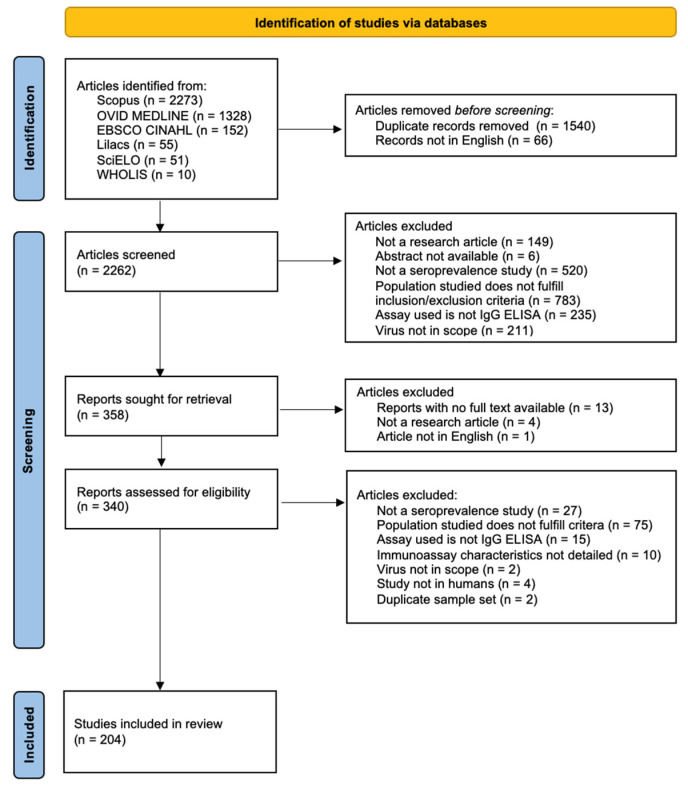
PRISMA flow diagram.

The study selection process is shown in Figure 1. In this review, 3869 records were retrieved from six online databases. After the removal of duplicates from three databases of published literature and of articles not written in English, 2262 records remained. Of these, 358 were sought for retrieval, and 340 were assessed for eligibility. A total of 204 records were included in this systematic review after the selection process.

### 3.2. Distribution of Flavivirus Seroprevalence Studies

**Figure 2:**
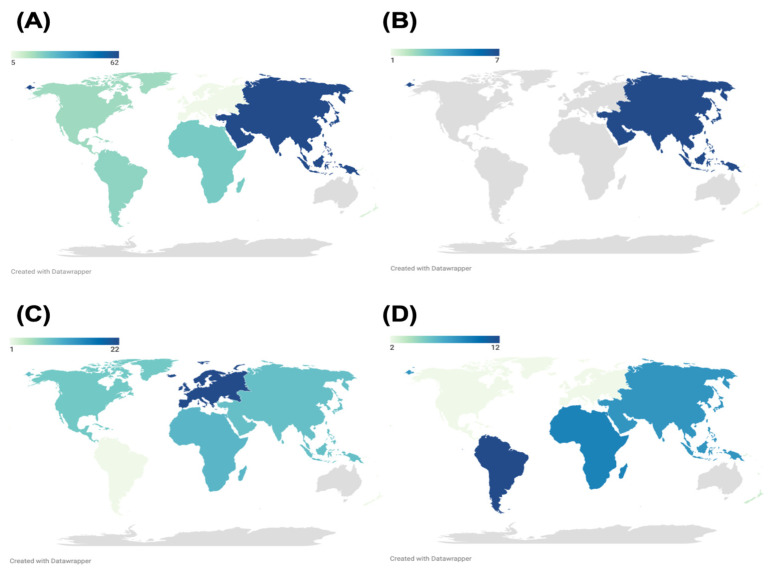
Distribution of flavivirus seroprevalence studies by region: (**A**) DENV, (**B**) JEV, (**C**) WNV, (**D**) ZIKV.

The results showed that serosurveys were most often performed for DENV (*n* = 136, 66.67%), followed by WNV (*n* = 50, 24.51%), ZIKV (*n* = 35, 17.16%), and JEV (*n* = 8, 3.92%). Commercial kits were also more commonly used (*n* = 159, 77.94%) than in-house assays (*n* = 48, 23.53%) overall. The indirect format was also most frequently used for flavivirus IgG ELISAs (*n* = 157, 76.96%), with a minority of studies performed using the antibody capture format (*n* = 22, 10.78%) and antigen capture format (*n* = 16, 7.84%) (Table 1). In terms of regional distribution, most of the seroprevalence studies overall were performed in Asia. About half of DENV serosurveys were performed in Asia (*n* = 62), followed by Africa (*n* = 23, 16.91%) and South America (*n* = 20, 14.71%), and almost all JEV studies were performed in Asia as well (*n* = 7, 87.5%) (Figure 2A,B). For WNV studies, the predominant region of origin was Europe (*n* = 22, 44%), whereas for ZIKV, it was South America (*n* = 12, 34.29%) (Figure 2C,D).

### 3.3. Types of ELISA and ELISA Antigens Used

**Figure 3:**
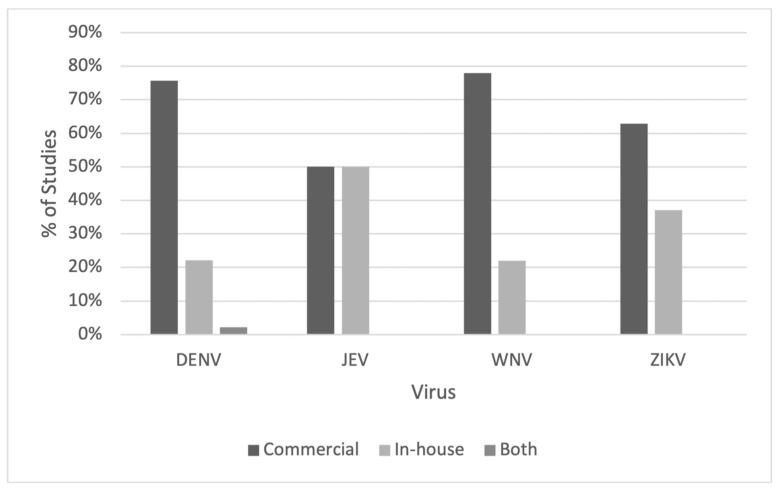
Distribution of ELISA types by virus.

**Figure 4:**
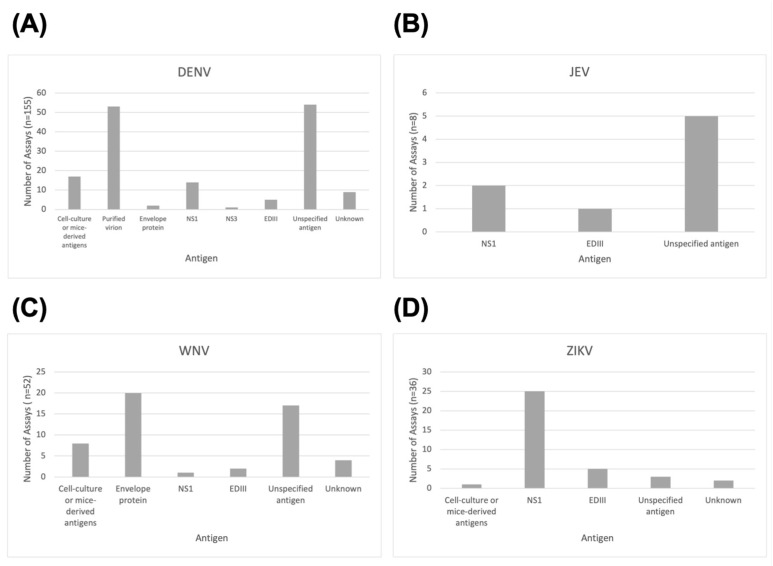
Distribution of ELISA antigens by virus: (**A**) DENV, (**B**) JEV, (**C**) WNV, (**D**) ZIKV.

For all viruses except JEV, commercial kits were more frequently used than in-house assays. A small number of studies for DENV (*n* = 3, 2.2%) used both commercial and in-house ELISAs (Figure 3). Figure 4A shows that the dominant antigens used for DENV assays are purified virions (*n* = 53, 34.19%) or unspecified antigens (*n* = 54, 34.84%). Most of these were from commercial test kits that do not detail how antigens are prepared for their ELISAs. This is also the case for JEV (Figure 4B), for which most studies with unspecified antigens used commercial assays. For ZIKV (Figure 4C), the NS1 antigen was most commonly used (*n* = 25, 69.44%), whereas for WNV (Figure 4D), 38.46% (*n* = 20) of the antigens used in ELISA assays included the envelope protein.

### 3.4. Serosurvey Trends over Time

**Figure 5:**
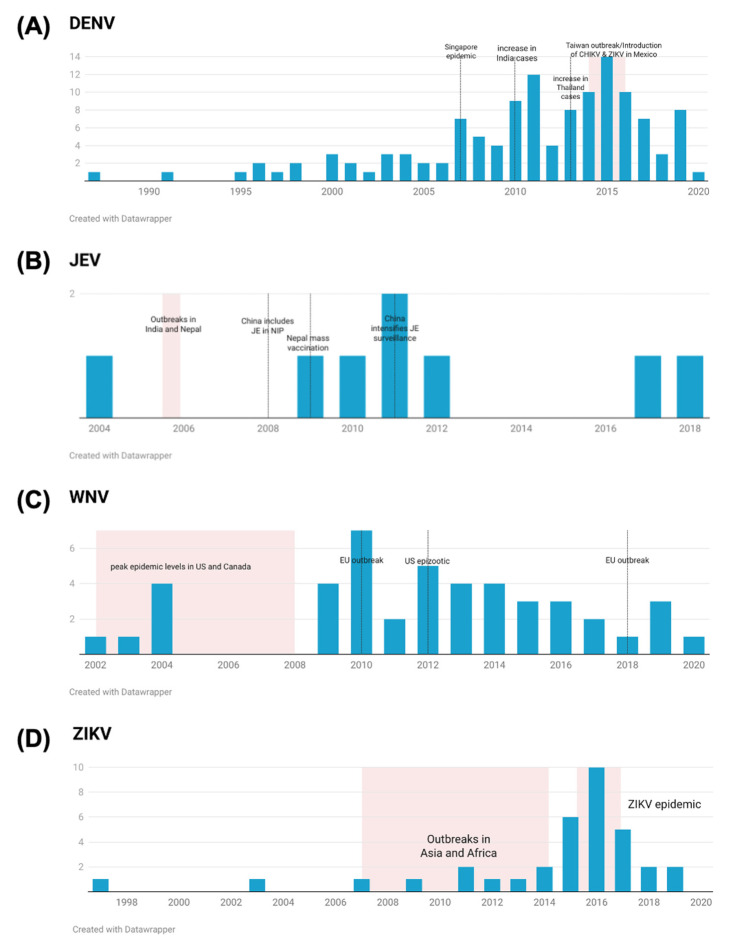
Trends in flavivirus seroprevalence studies over time: (**A**) DENV, (**B**) JEV, (**C**) WNV, (**D**) ZIKV.

It is shown in Figure 5 that for DENV, WNV, and ZIKV, the number of seroprevalence studies increased during or immediately after an outbreak or epidemic. This was not the case for JEV, for which seroprevalence studies were mainly performed to monitor vaccine response.

## 4. Discussion

Serosurveys are a direct measure of a population’s immunity to a specific pathogen, and when done routinely or periodically, they are known as serosurveillance [40]. This study determined trends in flavivirus IgG serosurveys for DENV, ZIKV, JEV, and WNV.

### 4.1. Regional Distribution of Flavivirus Serosurveys

Generally, the regional distribution of seroprevalence studies follows the trend of overall disease prevalence, which highlights the role of serosurveys in mapping disease burden. This in turn guides countries in the steps to be taken to ensure disease control. Among the four viruses, DENV was the most well studied. This is expected, as DENV is the most prevalent arthropod-borne virus globally, with cases steadily increasing over the past 70 years [3]. Most DENV studies were performed in Asia, consistent with the findings of one study that 70% of the disease burden is concentrated here [11]. This is because DENV is known to be widespread in tropical regions with the rainfall, temperature, urbanization, and prevalence of its mosquito vector proving to be suitable for transmission [41]. Similarly, the least number of studies were performed in Europe, where disease risk is also the lowest [42]. However, it can be expected that studies from this region would increase in the coming years, as globalization, international travel, and global warming threaten the emergence of DENV in previously non-endemic temperate regions [43]. A region in which disease occurrence does not match published data is Africa. Only 23 studies were contributed overall, despite a long history of DENV outbreaks in at least 16 countries in the region [44]. Other studies have similarly observed an underreporting of surveillance data from the region [11,44]. Many factors contribute to this, such as the lack of adequate surveillance systems, laboratory capabilities, as well as human and financial resources [44]. For ZIKV, most serosurveys were performed in South America, where ZIKV infections are clustered [45]. This was followed by Africa and Asia, where the virus has long been known to circulate [46]. The least number of studies were performed in Europe, where cases were mainly composed of travelers, until 2019, when the first autochthonous transmission was recorded in France [16]. Though ZIKV now causes more infections annually compared to WNV [3], there were more WNV seroprevalence studies performed, possibly owing to its earlier discovery [46,47] and with outbreaks occurring even before the re-emergence of ZIKV [20,48,49]. The regional distribution of WNV studies here also follows its known prevalence, which is mainly in North America, Europe, Africa, the Middle East, and West Asia [50]. Though 20% of the WNV studies included came from Africa, the region is still understudied, considering that WNV is present in 28 countries in the continent, with active viral circulation since the 1950s [51]. WNV is also known to be present in Australia [50]; however, no studies from the country were included in this study. Meanwhile, JEV had the lowest number of serosurveys, with the most studies conducted in Asia, since JEV is mainly distributed across this region but also has sporadic occurrences in northern Australia [52]. The low number of JEV studies is likely because it was the first to have a licensed vaccine among the four viruses [20,53,54,55], with cases markedly decreased after the start of vaccination campaigns [53]. Other possible reasons include the difficulty in detecting specific antibodies in healthy populations, such as anti-JEV NS1 antibodies, due to their low levels in subclinical infection [56] and the limited laboratory capacity in rural areas where the disease is most prevalent [53]. Thus, the use of IgG and IgM ELISAs has been largely limited to clinical diagnosis [57], with results not routinely reported by diagnostic laboratories.

### 4.2. Temporal Distribution of Flavivirus Serosurveys

Over time, it is observed that the number of serosurveys tends to fluctuate depending on the occurrence of outbreaks and epidemics for DENV, ZIKV, and WNV. The distribution of publications by country over time is shown in Table S2. For DENV, the 2007 epidemic in Singapore [58] and the concurrent rise in cases in other Southeast Asian countries [59] caused a spike in the number of publications, with three of seven of the included publications in that year coming from Southeast Asia. This is also the case for India, Thailand, and Taiwan, which had an increase in serosurveys published after cases increased in these respective countries [60,61,62]. In Mexico, DENV has been endemic for many years [63], and the trend observed was an increase in DENV serosurveillance after the introduction of related viruses such as ZIKV and chikungunya virus (CHIKV) in the country [64]. For JEV, the temporal distribution of studies did not follow the occurrences of outbreaks. JEV outbreaks and epidemics now rarely occur, largely due to vaccination programs [65]. Seven out of the eight JEV studies included here are from Asian countries. Of these, studies from countries with mass vaccination programs (i.e., China, Japan, South Korea, and Nepal) [52] were performed to monitor the effectiveness of such programs. Meanwhile, WNV was at its peak epidemic levels in Canada and the United States between 2002 and 2007 [47], and four of the seven studies included here were from these two countries. In 2010, an increase in serosurveys was noted, with four out of seven studies for that year coming from Europe. This is concurrent with an outbreak in Greece and increased viral circulation in other European countries [66,67]. Though Europe experienced its largest WNV outbreak in 2018 [48], no subsequent increase in IgG serosurveys among the general population was observed. There was also no noted increase in published IgG serosurveys from the United States after the 2012 epizootic in the country [49]. Lastly, the temporal distribution of ZIKV serosurveys was observed to be clustered around the 2015–2016 epidemic in South America, with almost half of the studies performed using sera from this period. Before this, the literature mostly came from African countries and some from Asia and French Polynesia, where a ZIKV outbreak occurred in 2013–2014 [46].

### 4.3. IgG ELISAs Used in Flavivirus Serosurveys

In this review, it was found that researchers used commercial kits more often, except for JEV, for which commercially available ELISAs and in-house assays were used equally. The benefits of using commercial kits for most non-endemic settings are obvious, since they are readily available and easy to use, and researchers need not develop or purchase their antigens and antibodies. Further, in-house assays need to be validated in terms of their diagnostic performance before use, whereas these data are usually already reported for commercial kits. However, there are still instances in which the use of in-house assays would be preferred. For example, in this study, the top countries/territories that used in-house ELISAs were Thailand, French Polynesia, and Brazil, which are all areas in which multiple flaviviruses are prevalent. In-house assays in these settings are advantageous, since they would be more cost-effective when testing a huge volume of sera. This may be the case in endemic areas where a large portion of the population is potentially affected. For these countries, seroprevalence testing may also be performed for multiple flaviviruses at the same time, so in-house assays would allow researchers to standardize the assay format and serum dilutions for all viruses to be tested against, allowing findings to be readily comparable. Further, the validation step for in-house assays is useful for determining an optimal cut-off value for the positive-to-negative (P/N) ratio. In areas in which multiple flaviviruses co-circulate, more cross-reactive antibodies in the sera are expected, and thus more background noise in ELISA assays, compared to sera from non-endemic regions. This means set cut-off values as used in commercial kits may not readily be applicable. This limitation could be addressed by using standard sera with known infection or vaccination histories for cut-off setting, but this is not routinely done, as commercial kits often come with packaged controls. In-house assays used in surveillance also benefit from quality assurance performed by national or reference centers to ensure protocol standardization as recommended by the WHO [68].

Among commercial kits used for DENV studies, the PanBio Dengue IgG Indirect ELISA was the most frequently used brand (Table S3). It is reported to have a sensitivity of 97.9% for secondary infections, a sensitivity of 62.0% for endemic samples, and a specificity of 100.0% [69]. Its high sensitivity and specificity as well as its wide availability are possibly what make it popular among researchers. However, its use in endemic regions may be limited based on its reported diagnostic performance. For ZIKV, the Euroimmun Anti-ZIKV IgG ELISA was the most common (Table S3). The manufacturer reports a sensitivity of 100.0% and a specificity of 97.0% using ZIKV RT-PCR-positive sera from endemic countries as samples and DENV-positive sera from Germany as controls [70]. They also report results of cross-reactivity testing against other flaviviruses such as DENV, JEV, WNV, yellow fever virus (YFV), and tick-borne encephalitis virus (TBEV) [70]. Other than a 0.7% cross-reactivity to DENV and a 4% cross-reactivity to JEV, no other false positives were detected [70]. This high diagnostic performance has similarly been confirmed by other studies comparing the Euroimmun ZIKV IgG ELISA to other commercially available brands [71,72]. For WNV, two test kits stood out in terms of frequency of use in studies: Euroimmun and Focus Diagnostics IgG ELISAs (Table S3). According to testing performed by the Food and Drug Administration (FDA) in 2003, the Focus Diagnostics kit has a sensitivity of 36.0–100.00% using samples from WNV patients or those that are WNV positive on PRNT [73]. Meanwhile, it correctly classified 96.8% of WNV negative samples in one study site [73]. However, its reported cross-reactivity with DENV is as high as 95% and ranges from 30.0–57.1% for other flaviviruses such as JEV, YFV, and the St. Louis encephalitis virus (SLEV) [73]. The Euroimmun kit, on the other hand, has a reported sensitivity of 91.1% and a specificity of 100.0% according to one study that used patient samples [74]. It is also reported to have low cross-reactivity to other flaviviruses [75]. This may be why for WNV serosurveys, Focus kits were more commonly used by earlier studies, but later studies have shifted towards a preference for Euroimmun kits (Table S4). JEV was the only virus for which a preference for commercial kits was not observed, since there is a relatively lower number of IgG ELISA kits available in the market. Other contributory factors include its lower prevalence and the difficulties in JEV IgG detection as previously discussed. Moreover, due to the low prevalence, JEV serology is usually applied only in point-of-care settings for disease diagnosis wherein IgM rather than IgG detection is performed [76].

### 4.4. ELISA Formats and Antigens in Flavivirus Serosurveys

For ELISA formats, the indirect method was more commonly used than sandwich methods (antibody capture or antigen capture). Indirect ELISA involves direct antigen coating onto an ELISA plate, followed by the sample sera that contains the antibody of interest, which is then detected by an enzyme-tagged secondary antibody [36]. Since IgG is the predominant antibody isotype in human sera [77], an indirect method would suffice in detecting its attachment, especially to an immunodominant antigen. This is as compared to other antibody isotypes such as IgM, which is lower in quantity and is more transient [77,78]. Thus, more sensitive methods such as the antibody capture format are needed to ensure its detection. A previous study has already shown that for IgM, the addition of an IgG depletion step is beneficial in increasing ELISA diagnostic performance [79]. However, sandwich formats are also utilized in IgG detection if the purpose is geared towards the detection of more recent infection [80]. This is based on the concept of avidity, since IgG antibodies are known to gain avidity over time [81] and would be less likely to detach during the washing step in ELISA. In an indirect format, this means IgG from past infection would be more likely to bind than those from more recent infection. In an antibody capture format, however, IgG antibodies would indiscriminately be bound by the capture antibody, regardless of avidity to the antigen of interest.

In terms of antigens, there were no temporal trends found in this study (Table S5). Rather, the choice of preferred antigen generally depended on the virus being studied. For DENV, predominant antigens were either purified DENV virions from serotypes 1 to 4 as used in Panbio kits [69] or other unspecified antigens from various commercial kits. When using virions, what would mostly be detected are anti-E antibodies, since these are the major target of the anti-flavivirus immune response [82]. This was also the antigen of choice for most WNV studies. The E protein is the major structural protein on the viral surface [83] and is known to be immunodominant [84], making it a clear antigen of choice. However, it has also been found that E-protein-based assays are prone to cross-reactivities [84] due to the protein’s high conservation and level of similarity among flaviviruses [85]. For ZIKV, NS1 was overwhelmingly preferred as an assay antigen. The NS1 protein has been found to be less cross-reactive and more specific than the E protein [84] while still being highly immunogenic [86]. This specificity is crucial for accurate ZIKV detection, as it is a virus that often co-circulates with related flaviviruses [87]. A small number of studies also made use of EDIII for all four viruses. EDIII is a distinct domain on the flavivirus E protein that has recently been of interest due to the presence of epitopes recognized by potent neutralizing antibodies [88], suggesting a potential role in flavivirus differentiation [89]. Meanwhile, most JEV studies merely indicated “JE recombinant antigen” or “purified JE antigen”, whereas others specified using either NS1 or EDIII.

### 4.5. Challenges in ELISA-Based Flavivirus Serosurveillance

This study shows that flavivirus serology is an evolving science with regional, temporal, and epidemiological factors that should be accounted for when conducting serosurveys. One major gap that this review highlights is the inconsistencies in assay use and reporting. A previous study has already pointed out that proper comparison of seroprevalence data is made challenging by the utilization of various commercial kits and in-house assays with a wide range of sensitivities and specificities [24]. Moreover, these assays differed in assay formats, antigens used, and detection systems [90]. Another challenge to study comparisons is that commercial brands do not report the specific antigen they coat onto their readymade ELISA plates for proprietary reasons. Further, in the present study, we found that the diagnostic performances of assays used are also not routinely reported, which makes adjusted analyses of seroprevalence rates difficult to perform. The authors suggest that researchers and peer reviewers alike should endeavor to include these data in future seroprevalence studies to allow for proper comparison. Studies are also not routinely conducted or reported, since the papers followed a temporal pattern based on the occurrence of outbreaks and epidemics. This highlights gaps in what is known about flavivirus immunity when not in peak disease season, which can be explored in future studies, as this is especially important in endemic areas where there is constant viral circulation.

### 4.6. Limitations of the Study

The findings of this review are limited to four medically important flaviviruses and to IgG seroprevalence studies performed on healthy populations using ELISA. Several other seroprevalence studies are performed for the purpose of mapping the occurrence of recent infection. These commonly make use of techniques that involve the detection of IgM or viral antigens, which is beyond the scope of the present study. This review is also limited to what is present in the published literature. The effect of economic constraints in limiting the number of studies performed or published in some countries cannot be discounted. There are also seroprevalence studies not included here, since they made use of other diagnostic tests such as neutralization assays, hemagglutination assays, or lateral flow immunoassays, used specimens other than sera, or detected other antibody isotypes. Further research is needed to understand the impact of these studies in the field of flavivirus epidemiology and serology.

## 5. Conclusions

This review shows how flavivirus epidemiology relates to the regional and temporal distribution of serosurveys. The role of factors such as endemicity, virus cross-reactivities, commercial kit availability, and study purpose in the choice of assay used were also shown. Gaps in detailing assay methodologies in serosurveys must be addressed, so that each study’s assay choice and reported seroprevalence can be adequately evaluated in the future.

## Figures and Tables

**Table 1:** Distribution of flavivirus seroprevalence studies (*n* = 204).

Category	Frequency	Percentage
Virus		
DENV	114	55.88%
JEV	6	2.94%
WNV	39	19.12%
ZIKV	23	11.27%
Multiple viruses	22	10.78%
*DENV and JEV*	*1*	*0.49%*
*DENV and WNV*	*9*	*4.41%*
*DENV and ZIKV*	*10*	*4.90%*
*DENV, WNV, and ZIKV*	*1*	*0.49%*
*DENV, JEV, WNV, and ZIKV*	*1*	*0.49%*
ELISA Type		
Commercial	156	76.47%
In-house	45	22.06%
Commercial and in-house	3	1.47%
ELISA Format		
Indirect	143	70.10%
Antibody capture	16	7.84%
Antigen capture	15	7.35%
Others	6	2.94%
Unspecified	10	4.90%
Multiple formats	14	6.86%
*Indirect and antibody capture*	*6*	*2.94%*
*Indirect and antigen capture*	*1*	*0.49%*
*Indirect and others*	*3*	*1.47%*
*Indirect and unspecified*	*4*	*1.96%*

## Data Availability

The data presented in this study are available in the article and its Supplementary Materials.

## Supplementary Materials

The following supporting information can be downloaded at: https://www.mdpi.com/article/10.3390/tropicalmed8040224/s1, Table S1: Quality assessment of included articles; Table S2: Distribution of countries by start year of serum collection for each virus; Table S3: Distribution of commercial ELISA kits used per virus; Table S4: Temporal distribution of WNV studies that used Euroimmun vs. Focus Diagnostics ELISA kits; Table S5: Temporal distribution of antigens used for each virus.
